# Supplementary material for: The homeodomain regulates stable DNA binding of prostate cancer target ONECUT2
Source: Nat Commun. 2024 Oct 19;15:9037. doi: 10.1038/s41467-024-53159-8 (PMC11490551; doi:10.1038/s41467-024-53159-8)
Supplement: Supplementary file 1 — Supplementary Information [file 41467_2024_53159_MOESM1_ESM.pdf]

a

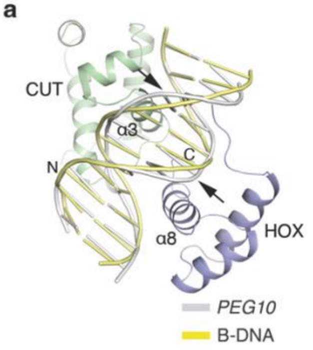

b

PEG10 sequence	Major groove *PEG10* (Å)	Major groove B-DNA (Å)	Minor groove *PEG10* (Å)	Minor groove B-DNA (Å)
AT/AT	18.5	17.3	10.9	13.8
TC/GA	16.5	17.3	13.3	12.0
CG/CG	17.5	17.5	13.6	10.5
GA/TC	20.3	16.8	10.9	9.9
AT/AT	18.0	17.4	9.3	9.3
TT/AA	17.5	18.5	10.1	10.0
TT/AA	16.8	17.8	10.8	11.0

c **d** **e**

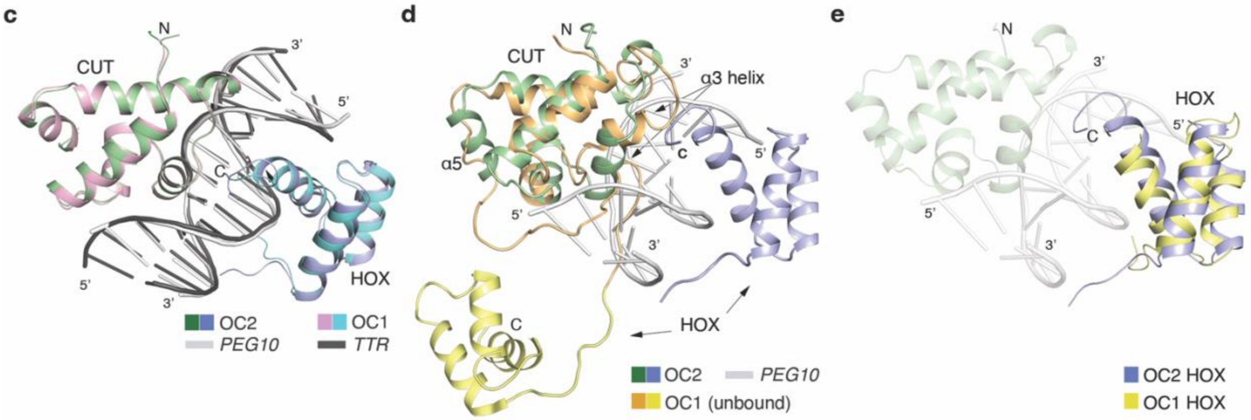

f

PEG10 DNA sequence:

5'- T A_1 G_2 A_3 T_4 C_5 G_6 A_7 T_8 T_9 T_{10} G_{11} C_{12} A -3'

3'- A $T_{1'}$ $C_{2'}$ $T_{3'}$ $A_{4'}$ $G_{5'}$ $C_{6'}$ $T_{7'}$ $A_{8'}$ $A_{9'}$ $A_{10'}$ $C_{11'}$ $G_{12'}$ T -5'

Supplementary Figure 1. Structural analyses of OC2-*PEG10* complex. (a) Structural alignment of OC2-bound *PEG10* DNA and a canonical B-DNA. The minor distortion in the OC2-bound *PEG10* DNA major groove, relative to the normal B-DNA, is marked with arrows. **(b)** The major and minor groove distances between phosphate backbones of complimentary bases are shown for both *PEG10* and the B-DNA. Distances are in angstrom units (Å). **(c)** Alignment of OC2-*PEG10* and OC1-*TTR* (PDB 2D5V[1] [https://doi.org/10.2210/pdb2D5V/pdb]) structures. The structures align with an rmsd of 0.78 Å. **(d)** Relative orientations of OC2 and OC1 in DNA bound and unbound (PDB 1S7E[2] [https://doi.org/10.2210/pdb1S7E/pdb]) states, respectively, with the CUT domains from the two structures aligned. The respective α3 helices are labeled. The CUT domains align with a rmsd of 3.3 Å while the α3 helix undergoes a rotation and structuring upon binding the DNA **(e)** Alignment of the HOX domains of the DNA bound OC2 and DNA unbound OC1. **(f)** The 14mer *PEG10* target DNA sequence for OC2 binding is shown. The 12mer sequence used in this study is shown in black, and numbered accordingly, with the excluded base pairs in grey. The N- and C-termini of the structures are labeled as also the 3' and 5' of the DNA.

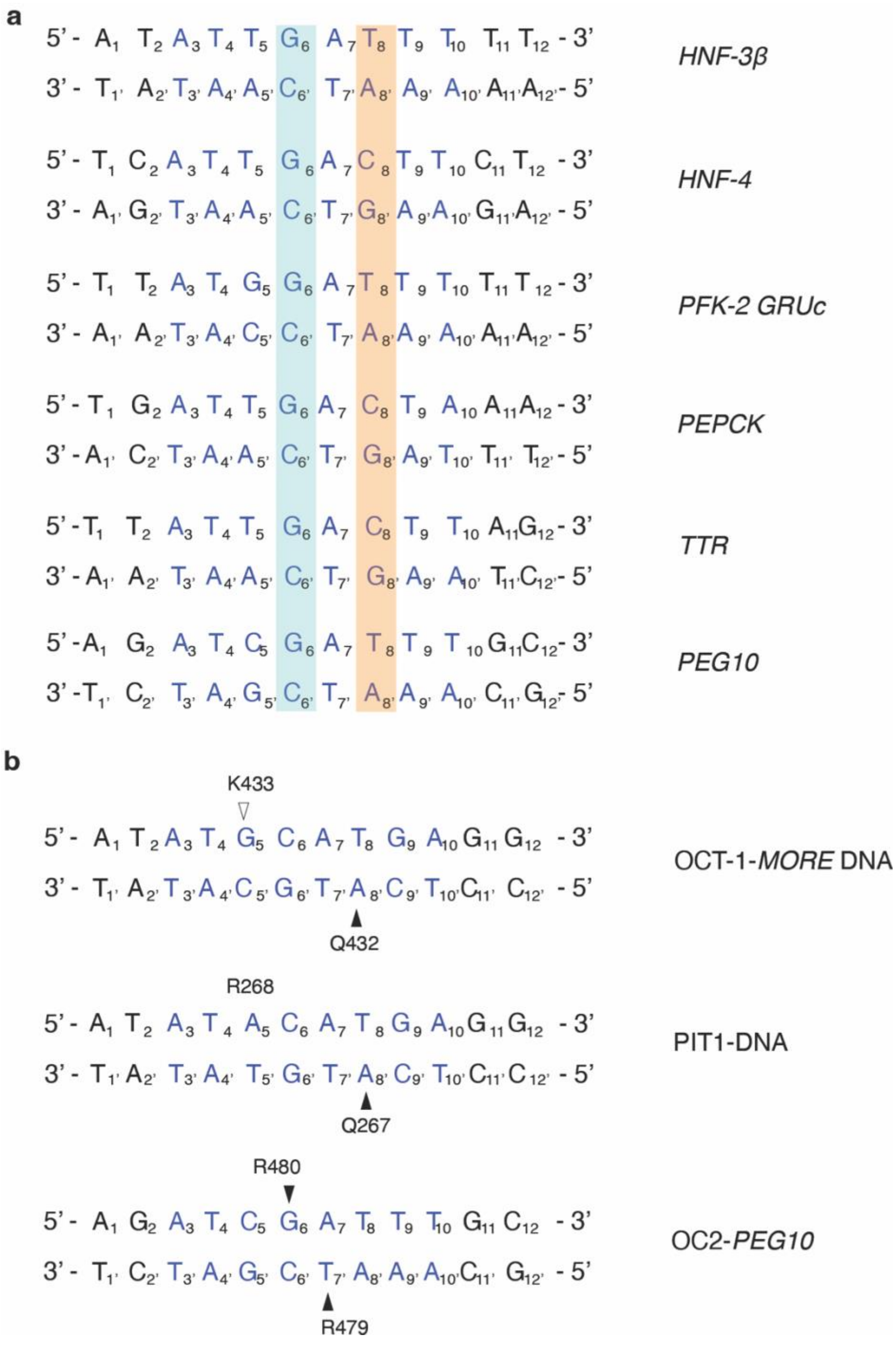

Supplementary Figure 2. OC and POU target promoter sequence analyses. **(a)** Common OC target promoter sequences, *HNF-3β* (hepatocyte nuclear factor-3β, *HNF-4* (hepatocyte nuclear factor-4), *PFK-2 GRUc* (6-phosphofructo-2-kinase glucocorticoid response unit c site), *PEPCK* (phosphoenolpyruvate carboxykinase) and *TTR* (transthyretin)[3]. The *PEG10* sequence is also shown for reference. Conservation of the guanine base at position 6, bound by OC2 R480 (or OC1 R439), is highlighted in cyan. The variable base at position 8 is highlighted in orange. **(b)** Sequence of DNA bound to OCT1 (*MORE* DNA; PDB 1E3O[4] [https://doi.org/10.2210/pdb1E3O/pdb]) and

PIT1 (PDB 1AU7[5] [https://doi.org/10.2210/pdb1AU7/pdb]) (as labeled on the right). The OC2 bound *PEG10* sequence is also shown (bottom) for reference. Note the conserved adenines at position 8' in OCT1 and PIT1 bound DNA that interacts with the conserved glutamine (OCT1 Q432 and PIT1 Q267; shown with solid black triangles) and the difference in position 6 of OC and POU bound DNA sequences. Interaction of OCT1 K433 to DNA backbone carbonyl group is depicted with an open black triangle. PIT1 R268 does not show any interaction in the corresponding structure.

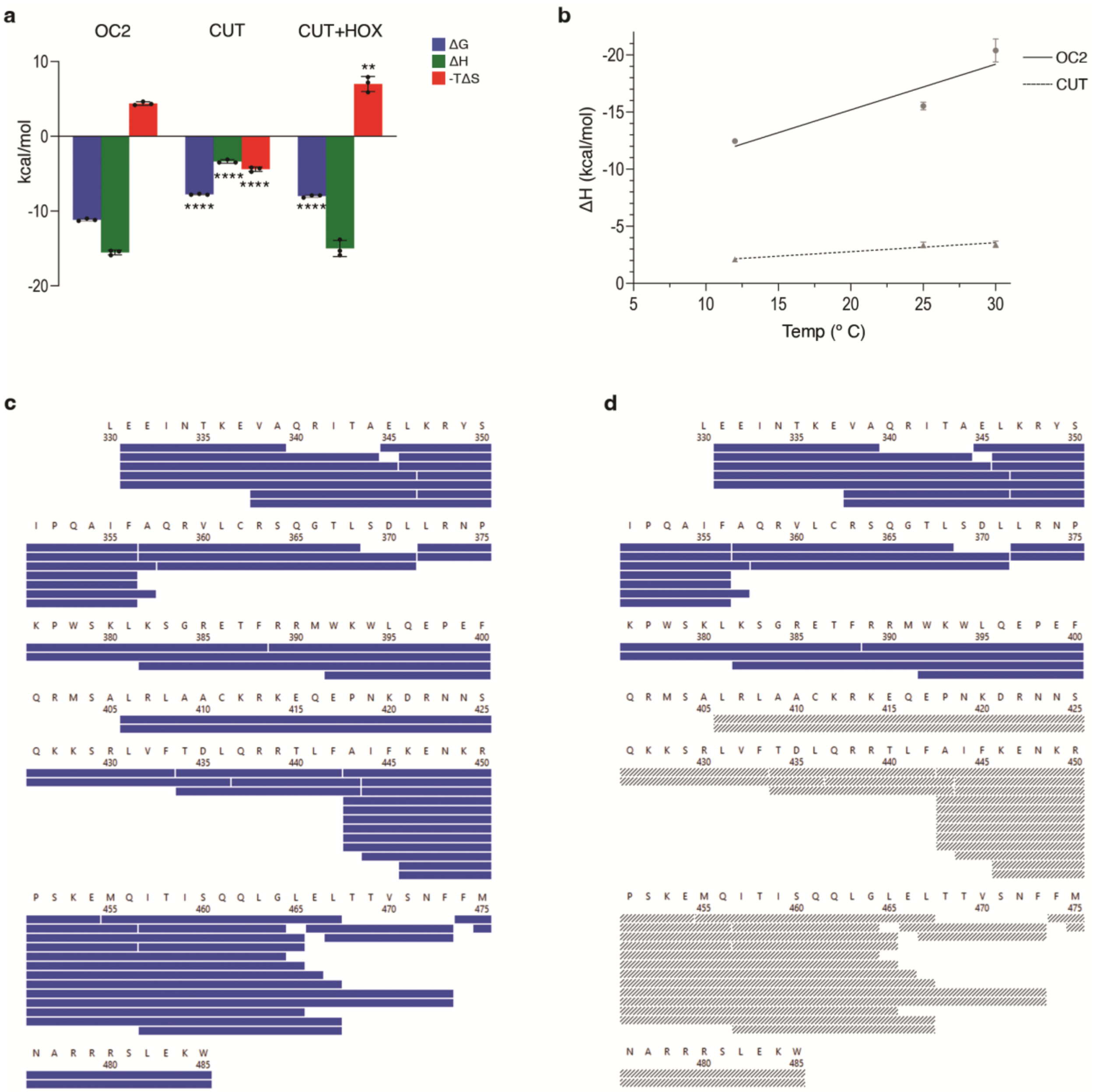

Supplementary Figure 3. Analyses of OC2-*PEG10* binding. (a) Thermodynamic signatures of binding of OC2, CUT and CUT+HOX to the PEG10 DNA. Data are presented as mean values ± SD (n=3; technical replicates). One-way ANOVA was used for statistical analysis. For ΔG, ****P(CUT)<0.0001 and ****P(CUT+HOX)<0.0001; for ΔH, ****P(CUT)<0.0001, P(CUT+HOX)=0.55; and for -TΔS, ****P(CUT)<0.0001, **P(CUT+HOX)=0.004. **(b)** Variation of ΔH with temperature. ΔH values calculated at 12, 25 and 30 °C for OC2-PEG10 (solid circles) and CUT-PEG10 (solid triangles) interactions are shown. Data are presented as mean values ± SD. Linear regression fitting of the points for OC2 and CUT are depicted as solid and dotted black lines, respectively. Source data for a-b are provided as Source Data files. **(c-d)** Coverage map of OC2 (c) and CUT (d) showing peptides present in each dataset and their relationship to one another. Peptides denoted in blue are present in the respective dataset, while cross-hatched bars

denote peptides that are present in OC2 but missing from CUT. The number of peptides, percent coverage of the entire sequence and peptide redundancy are listed in Supplementary Table 2.

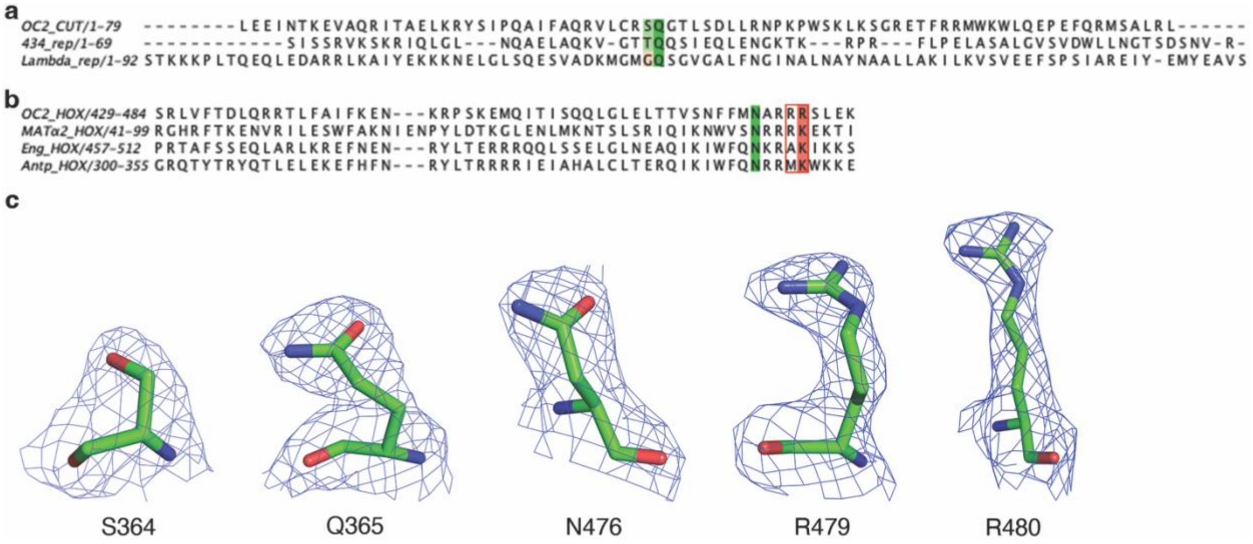

Supplementary Figure 4. Analysis of base-specific interacting residues of OC2. (a-b) Structure-based sequence alignment of DNA bound OC2 CUT, 434 phage repressor (PDB 2OR1 [https://doi.org/10.2210/pdb2OR1/pdb])[6], and Lambda phage repressor (PDB 1LMB [https://doi.org/10.2210/pdb1LMB/pdb])[7] domains (a) and homeodomains (HOX) of OC2, yeast MATα2 (PDB 1APL [https://doi.org/10.2210/pdb1APL/pdb])[8], *Drosophila* Engrailed (Eng) (PDB 1HDD [https://doi.org/10.2210/pdb1HDD/pdb])[9] and Antennapedia (Antp) (PDB 9ANT [https://doi.org/10.2210/pdb9ANT/pdb])[10] (b). The amino acid ranges for respective sequences are indicated. The conserved serine and glutamine of CUT, and asparagine of HOX, are shown with a color background. The arginine motif in OC2 HOX and corresponding residues in other HOX domains are shown with a red boundary. The background colors are in Clustal scheme and according to their conservation among the protein sequences shown. **(c)** Electron densities (2Fo-Fc map contoured at 1.0 σ) of the residues as observed in the OC2-*PEG10* complex structure.

a

Interaction	K_D (nM)	ΔH (kcal/mol)	ΔG (kcal/mol)	-TΔS (kcal/mol)	N (sites)
OC2R479 - *PEG10*	6 ± 2	-15.9 ±0.2	-11.2 ±0.2	4.7 ±0.4	0.8 ±0.1
OC2R480 - *PEG10*	47 ± 7	-13.4 ±0.4	-10.0 ±0.1	3.4 ±0.4	0.8 ±0.2

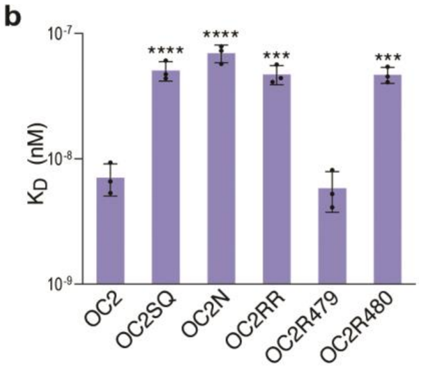

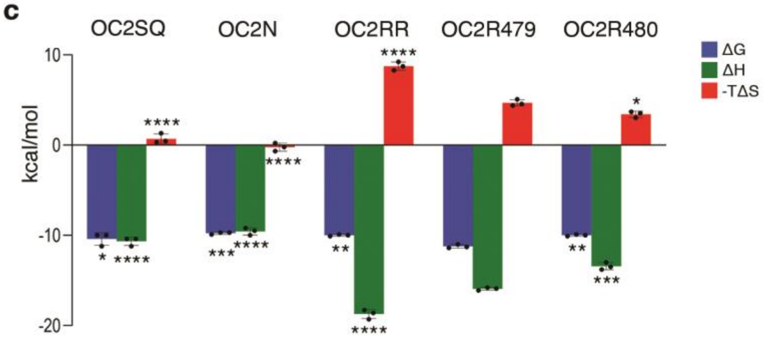

Supplementary Figure 5. ITC binding analysis of base-specific mutants of OC2. (a) Thermodynamics parameters of interaction between OC2R479A and OC2R480A single mutants to the *PEG10* DNA. Data are presented as mean values ± SD (n=3; technical replicates). **(b)** Comparison of binding affinity (K_D) between wild-type and mutant OC2 proteins to *PEG10* DNA. One-way ANOVA was used for statistical analysis. ****P(OC2SQ)<0.0001, ****P(OC2N)<0.0001, ***P(OC2RR)=0.0001, P(OC2R479)=0.99, ***P(OC2R480)=0.0001. **(c)** Thermodynamic signatures of binding of wild-type and mutant OC2 proteins to the *PEG10* DNA. Respective parameters between OC2 and each mutant was analyzed. One-way ANOVA was used for statistical analysis. For ΔG, *P(OC2SQ)=0.04, ***P(OC2N)=0.0006, **P(OC2RR)=0.003, P(OC2R479)=0.99, **P(OC2R480)=0.003; for ΔH, ****P(OC2SQ)<0.0001, ****P(OC2N)<0.0001, ****P(OC2RR)<0.0001, P(OC2R479)=0.62, ***P(OC2R480)=0.0001; and for -TΔS, ****P(OC2SQ)<0.0001, ****P(OC2N))<0.0001, ****P(OC2RR))<0.0001, P(OC2R479)=0.85, *P(OC2R480)=0.05. Source data are provided as a Source Data file.

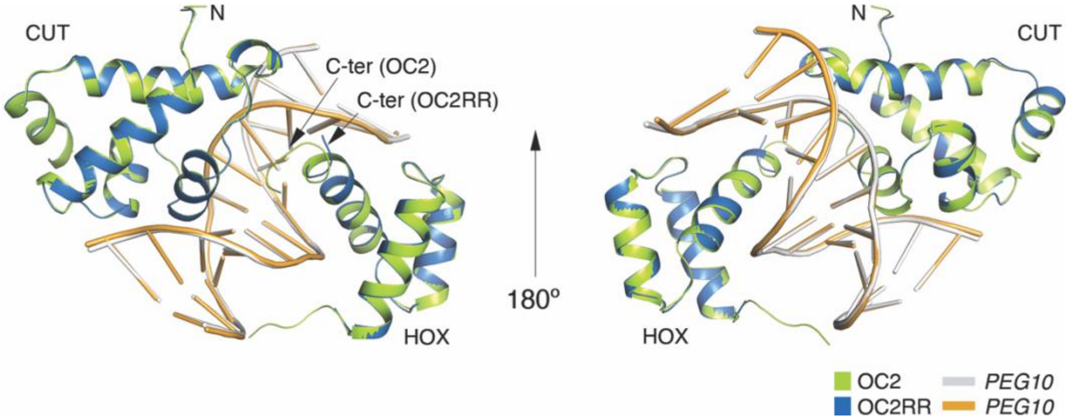

Supplementary Figure 6. Structural alignment of OC2 and OC2RR mutant. The respective structures align with a rmsd of 0.296 Å. The CUT and HOX domains are labeled. The N-terminal is also labeled while the C-terminal of both structures are marked with arrows in the left panel.

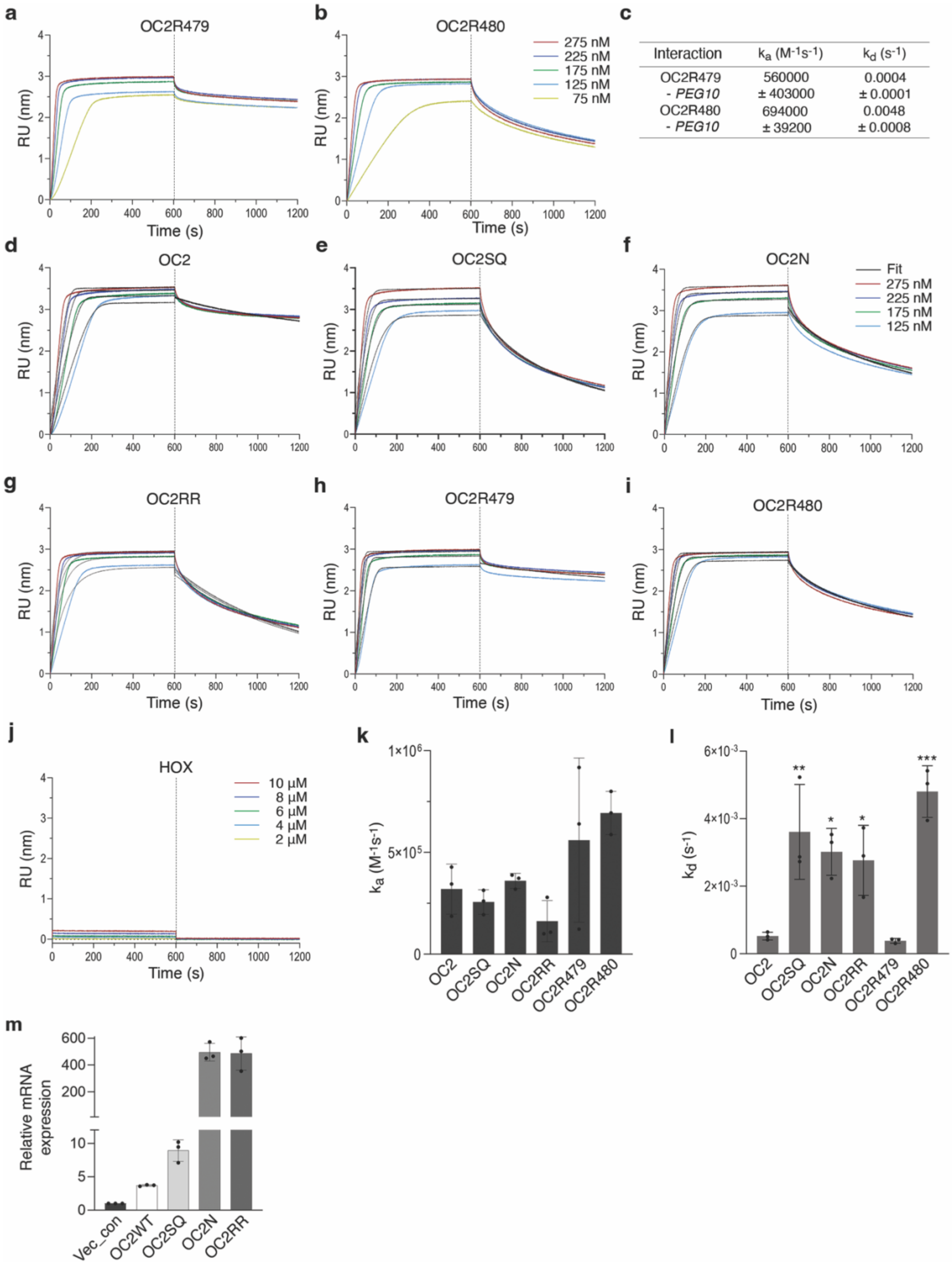
a
OC2R479
RU (nm)
Time (s)
b
OC2R480
275 nM
225 nM
175 nM
125 nM
75 nM
RU (nm)
Time (s)
c
Interaction
k_a ($M^{-1}s^{-1}$)
k_d (s^{-1})
OC2R479 - *PEG10*
560000 ± 403000
0.0004 ± 0.0001
OC2R480 - *PEG10*
694000 ± 39200
0.0048 ± 0.0008
d
OC2
e
OC2SQ
f
OC2N
Fit
275 nM
225 nM
175 nM
125 nM
g
OC2RR
h
OC2R479
i
OC2R480
RU (nm)
Time (s)
j
HOX
10 μM
8 μM
6 μM
4 μM
2 μM
RU (nm)
Time (s)
k
k_a ($M^{-1}s^{-1}$)
OC2
OC2SQ
OC2N
OC2RR
OC2R479
OC2R480
l
k_d (s^{-1})
**
*
*
***
OC2
OC2SQ
OC2N
OC2RR
OC2R479
OC2R480
m
Relative mRNA expression
Vec_con
OC2WT
OC2SQ
OC2N
OC2RR

Supplementary Figure 7. Binding kinetics and functional analyses of the OC2 mutants. (a-c) Binding kinetics of R479A (a) and R480A (b) mutants to the *PEG10* DNA, and their rates of association and dissociation (c). The concentrations 75, 125, 175, 225 and 275 nM, and representative curves are shown (n=3; technical replicates). **(d-i)** Representative association and dissociation kinetics curves with respective fits for wild-type and mutant OC2 proteins (125, 175, 225 and 275 nM concentrations used for kinetics analyses). **(j)** Binding kinetics of the isolated HOX domain to the *PEG10* DNA. **(k-l)** Comparison of rates of association (k) and dissociation (l) of wild-type OC2 to that of the mutants. Data are presented as mean values ± SD (n=3; technical replicates). One-way ANOVA was used for statistical analysis. For rate of association (k_a), P(OC2SQ)=0.99, P(OC2N)= 0.99, P(OC2RR)=0.75, P(OC2R479)=0.40, P(OC2R480)=0.10; and for rate of dissociation (k_d), **P(OC2SQ)=0.003, *P(OC2N)= 0.01, *P(OC2RR)=0.02, P(OC2R479)=0.99, ***P(OC2R480)=0.0002. **(m)** Relative mRNA levels of endogenous *OC2* (Vec_con), *OC2 wild-type (OC2WT)*, *OC2SQ*, *OC2N* and *OC2RR*, in respective LNCaP cells. Source data (a-l) are provided as a Source Data file.

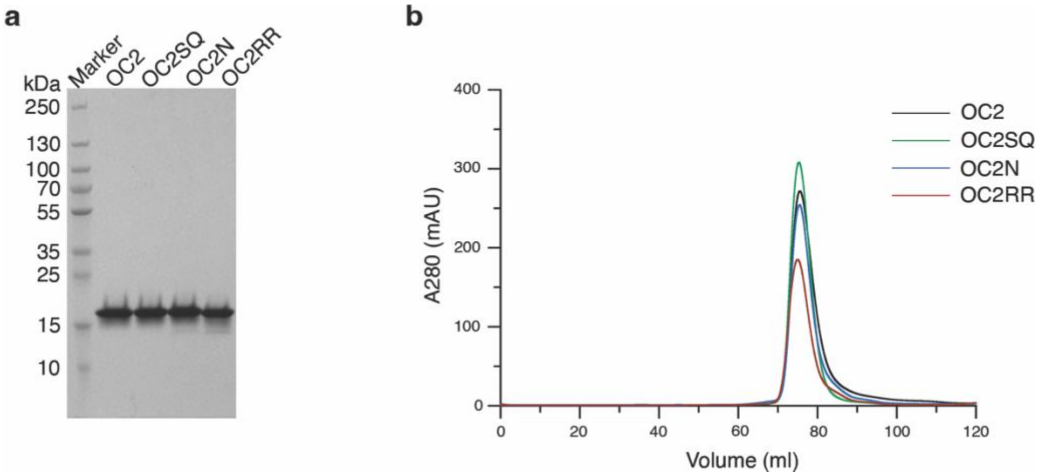

Supplementary Figure 8. Purification profiles of OC2 and its mutants. (a) SDS-PAGE gel showing profiles of all purified proteins. The molecular weights of marker bands are shown in kDa. **(b)** Gel-filtration (Superdex 200 16/600) profiles of indicated proteins are shown.

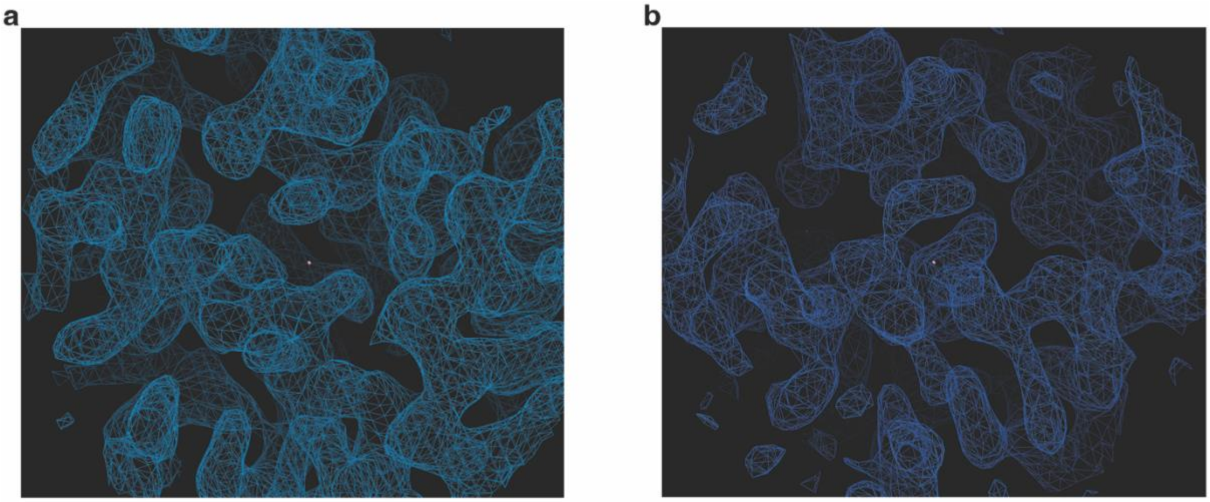

Supplementary Figure 9. Electron density maps. Part of 2Fo-Fc electron density maps contoured at 1.0 σ for **(a)** OC2-*PEG10* and **(b)** OC2RR-*PEG10* complex structures.

Supplementary Table 1

OC2-*PEG10* and OC2RR-*PEG10* crystallographic data collection and refinement statistics

**Parameter**	**OC2-*PEG10***	**OC2RR-*PEG10***
PDB code	8T0F	8T11
Wavelength (Å)	1.5418	1.5418
Resolution range (Å)	28.39 - 2.61 (2.703 - 2.61)	27.87 - 2.91 (3.014 - 2.91)
Space group	C 1 2 1	C 1 2 1
Unit cell parameters		
a, b, c (Å)	96.23, 78.30, 39.45	96.14, 78.15, 39.50
α, β, γ (°)	90, 111, 90	90, 111.44, 90
Total reflections	60782 (6008)	21153 (2129)
Unique reflections	8316 (818)	5960 (582)
Multiplicity	7.3 (7.3)	3.5 (3.7)
Completeness (%)	98.56 (98.20)	97.38 (95.65)
Mean I/sigma(I)	21.89 (3.06)	11.81 (1.61)
Wilson B-factor ($Å^2$)	52.34	56.44
R-merge	0.12 (1.12)	0.16 (1.28)
R-meas	0.13 (1.20)	0.19 (1.49)
CC1/2	0.999 (0.712)	0.994 (0.73)
CC*	1 (0.912)	0.998 (0.919)
Reflections used in refinement	8269 (818)	5881 (572)
Reflections used for R-free	431 (51)	434 (43)
R-work	0.2290 (0.3531)	0.2511 (0.4423)
R-free	0.2835 (0.4851)	0.3058 (0.6158)
CC (work)	0.953 (0.729)	0.940 (0.640)
CC (free)	0.928 (0.553)	0.902 (0.604)
Number of non-hydrogen atoms		
Total	1668	1535
Macromolecules	1631	1532
Ligands	0	0
Solvent	37	3
Protein residues	135	126
rmsd from ideal		
Bond lengths (Å)	0.004	0.003
Bond angles (°)	0.61	0.57
Ramachandran Plot		
Favored (%)	96.95	98.36
Allowed (%)	3.05	1.64
Outliers (%)	0.00	0.00
Rotamer outliers (%)	0.00	0.00
Clashscore	7.13	4.88
Average B-factor ($Å^2$)		
Overall	55.57	45.77
Macromolecules	60.86	48.78
Solvent	52.08	31.28

Values in parentheses are for the highest resolution shells

Supplementary Table 2

OC2- and CUT-DNA binding analyses using HDX-MS

	**OC2 apo**	**OC2+DNA**
HDX reaction details	10 mM Na_2HPO_4, 1.8 mM KH_2PO_4, 137 mM NaCl, 2.7 mM KCl, 0.5 mM TCEP, pD= 7.75@4 °C	10 mM Na_2HPO_4, 1.8 mM KH_2PO_4, 137 mM NaCl, 2.7 mM KCl, 0.5 mM TCEP, pD= 7.75@4 °C
HDX time course (min)	0.25, 0.5, 1, 2	0.25, 0.5, 1, 2
HDX control samples	Disordered termini of varied proteins	Disordered termini of varied proteins
Back-exchange (mean / IQR)	27%/5%	27%/5%
# of Peptides	45	45
Sequence coverage	96.2%	96.2%
Average peptide length / Redundancy	15.8/ 4.75	15.8/ 4.75
Replicates (biological or technical)	3 (technical)	3 (technical)
Repeatability	0.062 (average standard deviation)	0.082 (average standard deviation)
Significant differences in HDX (delta HDX > X D)	0.25D (99% CI)	0.25D (99% CI)

	**CUT apo**	**OC2+DNA**
HDX reaction details	10 mM Na_2HPO_4, 1.8 mM KH_2PO_4, 137 mM NaCl, 2.7 mM KCl, 0.5 mM TCEP, pD= 7.75@4 °C	10 mM Na_2HPO_4, 1.8 mM KH_2PO_4, 137 mM NaCl, 2.7 mM KCl, 0.5 mM TCEP, pD= 7.75@4 °C
HDX time course (min)	0.25, 0.5, 1, 2	0.25, 0.5, 1, 2
HDX control samples	Disordered termini of varied proteins	Disordered termini of varied proteins
Back-exchange (mean / IQR)	27%/5%	27%/5%
# of Peptides	20	20
Sequence coverage	79.5%	79.5%
Average peptide length / Redundancy	14.0/ 4.16	14.0/ 4.16
Replicates (biological or technical)	3 (technical)	3 (technical)
Repeatability	0.067 (average standard deviation)	0.058 (average standard deviation)
Significant differences in HDX (delta HDX > X D)	0.25D (99% CI)	0.25D (99% CI)

Supplementary Table 3
Primer sequences

Protein	Primer Sequence
OC2	FP: 5’- tacttccaatccaatgcactggaagaaatcaacaccaaagagg -3’<br>RP: 5'- ttatccacttccaatgttattatcaccacttctcgacgctgcggcgccggg -3’
CUT	FP: 5'- tacttccaatccaatgcatcgtcctcatcgggctcgcag -3'<br>RP: 5'- ttatccacttccaatgttattatcattcttgctctttgcgtttgca -3'
HOX	FP: 5'- tacttccaatccaatgcaaaagacaggaacaattcccag -3'<br>RP: 5'- ttatccacttccaatgttattatcagctcagatcgtcttgccactt -3'
OC2SQ (in two steps)	i) OC2 S364A mutant<br>FP: 5'- agagagtcccctgcgcccggcacagcacc -3'<br>RP: 5'- ggtgctgtgccgggcgcaggggactctct -3'
	ii) OC2 Q365A in S364A plasmid<br>FP: 5’- ggagagagtccccgccgcccggcacagc -3'<br>RP: 5’- gctgtgccgggcggcggggactctctcc -3'
OC2N	FP: 5'- tgcggcgccgggccgccatgaagaagttgctgacg -3'<br>RP: 5'- cgtcagcaacttcttcatggcggcccggcgccgca -3'
OC2RR	FP: 5’- ccacttctccaggctggcggcccgggcgttcatgaag -3'<br>RP: 5'- cttcatgaacgcccgggccgccagcctggagaagtgg -3'
OC2R479	FP: 5'-cttctccaggctgcgtgcccgggcgttcatgaa-3'<br>RP: 5'-ttcatgaacgcccgggcacgcagcctggagaag-3'
OC2R480	FP: 5'-cttctccaggctggcgcgccgggcgttc-3'<br>RP: 5'-gaacgcccggcgcgccagcctggagaag-3'

Supplementary References

1. Iyaguchi, D., Yao, M., Watanabe, N., Nishihira, J. & Tanaka, I. DNA recognition mechanism of the ONECUT homeodomain of transcription factor HNF-6. *Structure* **15**, 75-83 (2007).
2. Sheng, W., Yan, H., Rausa, F.M., 3rd, Costa, R.H. & Liao, X. Structure of the hepatocyte nuclear factor 6alpha and its interaction with DNA. *J Biol Chem* **279**, 33928-36 (2004).
3. Jacquemin, P., Lannoy, V.J., Rousseau, G.G. & Lemaigre, F.P. OC-2, a novel mammalian member of the ONECUT class of homeodomain transcription factors whose function in liver partially overlaps with that of hepatocyte nuclear factor-6. *J Biol Chem* **274**, 2665-71 (1999).
4. Remenyi, A. et al. Differential dimer activities of the transcription factor Oct-1 by DNA-induced interface swapping. *Mol Cell* **8**, 569-80 (2001).
5. Jacobson, E.M., Li, P., Leon-del-Rio, A., Rosenfeld, M.G. & Aggarwal, A.K. Structure of Pit-1 POU domain bound to DNA as a dimer: unexpected arrangement and flexibility. *Genes Dev* **11**, 198-212 (1997).
6. Aggarwal, A.K., Rodgers, D.W., Drottar, M., Ptashne, M. & Harrison, S.C. Recognition of a DNA operator by the repressor of phage 434: a view at high resolution. *Science* **242**, 899-907 (1988).
7. Beamer, L.J. & Pabo, C.O. Refined 1.8 A crystal structure of the lambda repressor-operator complex. *J Mol Biol* **227**, 177-96 (1992).
8. Wolberger, C., Vershon, A.K., Liu, B., Johnson, A.D. & Pabo, C.O. Crystal structure of a MAT alpha 2 homeodomain-operator complex suggests a general model for homeodomain-DNA interactions. *Cell* **67**, 517-28 (1991).
9. Kissinger, C.R., Liu, B.S., Martin-Blanco, E., Kornberg, T.B. & Pabo, C.O. Crystal structure of an engrailed homeodomain-DNA complex at 2.8 A resolution: a framework for understanding homeodomain-DNA interactions. *Cell* **63**, 579-90 (1990).
10. Fraenkel, E. & Pabo, C.O. Comparison of X-ray and NMR structures for the Antennapedia homeodomain-DNA complex. *Nat Struct Biol* **5**, 692-7 (1998).
