# Supplementary material for: The homeodomain regulates stable DNA binding of prostate cancer target ONECUT2
Source: Nat Commun. 2024 Oct 19;15:9037. doi: 10.1038/s41467-024-53159-8 (PMC11490551; doi:10.1038/s41467-024-53159-8)
Supplement: Supplementary file 3 — Reporting Summary [file 41467_2024_53159_MOESM3_ESM.pdf]

Corresponding author(s): Ramachandran Murali, Ph.D.

Last updated by author(s): Aug 16, 2024

Reporting Summary

Nature Portfolio wishes to improve the reproducibility of the work that we publish. This form provides structure for consistency and transparency in reporting. For further information on Nature Portfolio policies, see our Editorial Policies and the Editorial Policy Checklist.

Statistics

For all statistical analyses, confirm that the following items are present in the figure legend, table legend, main text, or Methods section.

n/a	Confirmed	
☐	☒	The exact sample size (*n*) for each experimental group/condition, given as a discrete number and unit of measurement
☐	☒	A statement on whether measurements were taken from distinct samples or whether the same sample was measured repeatedly
☐	☒	The statistical test(s) used AND whether they are one- or two-sided<br>*Only common tests should be described solely by name; describe more complex techniques in the Methods section.*
☒	☐	A description of all covariates tested
☒	☐	A description of any assumptions or corrections, such as tests of normality and adjustment for multiple comparisons
☐	☒	A full description of the statistical parameters including central tendency (e.g. means) or other basic estimates (e.g. regression coefficient) AND variation (e.g. standard deviation) or associated estimates of uncertainty (e.g. confidence intervals)
☐	☒	For null hypothesis testing, the test statistic (e.g. *F*, *t*, *r*) with confidence intervals, effect sizes, degrees of freedom and *P* value noted<br>*Give P values as exact values whenever suitable.*
☒	☐	For Bayesian analysis, information on the choice of priors and Markov chain Monte Carlo settings
☒	☐	For hierarchical and complex designs, identification of the appropriate level for tests and full reporting of outcomes
☒	☐	Estimates of effect sizes (e.g. Cohen's *d*, Pearson's *r*), indicating how they were calculated

Our web collection on statistics for biologists contains articles on many of the points above.

Software and code

Policy information about availability of computer code

Data collection	X-ray data - RIgaku Micromax 007<br>ITC data - Malvern PEAQ-ITC Control<br>BLI - Octet RED 384<br>HDXMS - Waters HDX-1
Data analysis	Phenix: 1.20.1<br>HKL-2000: v721.3<br>MOLREP: 11.7.03; 13.07.2020<br>COOT: v0.9.6<br>PyMol: 2.5.5<br>CCP4i: 8.0.012<br>SBGrid Consortium platform<br>PEAQ-ITC Analysis<br>TraceDrawer (Ridgeview Instruments): 1.9.2<br>ProteinLynx: global server (PLGS): v3.0; DECA: v116; DynamX v3.0.0<br>Clustal Omega; Jalview 2.11.3.3<br>GraphPad Prism 9.2.0<br>3DNA v2.4.3-2019apr06

For manuscripts utilizing custom algorithms or software that are central to the research but not yet described in published literature, software must be made available to editors and reviewers. We strongly encourage code deposition in a community repository (e.g. GitHub). See the Nature Portfolio guidelines for submitting code & software for further information.

Data

Policy information about availability of data

All manuscripts must include a data availability statement. This statement should provide the following information, where applicable:

- Accession codes, unique identifiers, or web links for publicly available datasets
- A description of any restrictions on data availability
- For clinical datasets or third party data, please ensure that the statement adheres to our policy

The PDB files of the structures with accession codes 8T0F [https://doi.org/10.2210/pdb8T0F/pdb] (OC2-PEG10) and 8T11 [https://doi.org/10.2210/pdb8T11/pdb] (OC2RR-PEG10) are available at wwpdb.org. HDXMS data is available at massive.ucsd.edu (Dataset MSV000094672). Source data are provided with this paper.

The following prior published structures used for analyses in this work are available at wwpdb.org: 2D5V [https://doi.org/10.2210/pdb2D5V/pdb] (OC1-TTR); 1S7E [https://doi.org/10.2210/pdb1S7E/pdb] (apo OC1); 1E3O [https://doi.org/10.2210/pdb1E3O/pdb] (OCT1); 1AU7 [https://doi.org/10.2210/pdb1AU7/pdb] (PIT1); 2OR1 [https://doi.org/10.2210/pdb2OR1/pdb] (434 phage repressor); 1LMB [https://doi.org/10.2210/pdb1LMB/pdb] (Lambda phage repressor); 1APL [https://doi.org/10.2210/pdb1APL/pdb] (yeast MAT 2); 1HDD [https://doi.org/10.2210/pdb1HDD/pdb] (Engrailed); 9ANT [https://doi.org/10.2210/pdb9ANT/pdb] (Antennapedia).

Research involving human participants, their data, or biological material

Policy information about studies with human participants or human data. See also policy information about sex, gender (identity/presentation), and sexual orientation and race, ethnicity and racism.

Reporting on sex and gender	n/a
Reporting on race, ethnicity, or other socially relevant groupings	n/a
Population characteristics	n/a
Recruitment	n/a
Ethics oversight	n/a

Note that full information on the approval of the study protocol must also be provided in the manuscript.

Field-specific reporting

Please select the one below that is the best fit for your research. If you are not sure, read the appropriate sections before making your selection.

☒ Life sciences ☐ Behavioural & social sciences ☐ Ecological, evolutionary & environmental sciences

For a reference copy of the document with all sections, see nature.com/documents/nr-reporting-summary-flat.pdf

Life sciences study design

All studies must disclose on these points even when the disclosure is negative.

Sample size	Experiments were performed in triplicates where applicable.
Data exclusions	No data were excluded.
Replication	Applicable experiments were repeated independently and were reproducible. The number of replicates have been noted in respective figure legends and Methods.
Randomization	Not relevant as our data mostly pertains to protein structural, biophysical and cell-based analyses.
Blinding	Not relevant to this study.

Reporting for specific materials, systems and methods

We require information from authors about some types of materials, experimental systems and methods used in many studies. Here, indicate whether each material, system or method listed is relevant to your study. If you are not sure if a list item applies to your research, read the appropriate section before selecting a response.

Materials & experimental systems

n/a	Involved in the study
☒	☐ Antibodies
☐	☒ Eukaryotic cell lines
☒	☐ Palaeontology and archaeology
☒	☐ Animals and other organisms
☒	☐ Clinical data
☒	☐ Dual use research of concern
☒	☐ Plants

Methods

n/a	Involved in the study
☒	☐ ChIP-seq
☒	☐ Flow cytometry
☒	☐ MRI-based neuroimaging

Eukaryotic cell lines

Policy information about cell lines and Sex and Gender in Research

Cell line source(s)	LNCaP cells (Cat# CRL-1740) from American Type Culture Collection (ATCC) were used.
Authentication	Cell lines were authenticated by STR (short tandem repeat) analysis.
Mycoplasma contamination	All cell lines tested negative for mycoplasma contamination.
Commonly misidentified lines (See ICLAC register)	No commonly misidentified cell lines were used.

Plants

Seed stocks	n/a
Novel plant genotypes	n/a
Authentication	n/a
